# Challenging the Concept of Uncomplicated Candidemia for Shorter Courses of Antifungal Therapy

**DOI:** 10.1093/ofid/ofag530

**Published:** 2026-08-22

**Authors:** Matthaios Papadimitriou-Olivgeris, Laurence Senn, Alix Coste, Frédéric Lamoth

**Affiliations:** Infectious Diseases Service, Lausanne University Hospital, Lausanne, Switzerland; Infectious Diseases Service, Institut Central des Hôpitaux and Hospital of Valais, Sion, Switzerland; Infectious Diseases Service, Lausanne University Hospital, Lausanne, Switzerland; Infection Prevention and Control Unit, Lausanne University Hospital, Lausanne, Switzerland; Institute of Microbiology, Lausanne University Hospital and University of Lausanne, Lausanne, Switzerland; Infectious Diseases Service, Lausanne University Hospital, Lausanne, Switzerland; Institute of Microbiology, Lausanne University Hospital and University of Lausanne, Lausanne, Switzerland

**Keywords:** candidemia, complicated candidemia, deep-seated infection, infectious diseases consultation, source control

## Abstract

**Background:**

A recent proposal introduced criteria to distinguish uncomplicated from complicated candidemia and identify patients who might be candidates for a shortened (7-day) course of antifungal therapy. We evaluated the feasibility and potential impact of this strategy in a real-world cohort.

**Methods:**

This retrospective study included patients with candidemia at Lausanne University Hospital (2015–2024). We estimated the sample size required for a randomized non-inferiority trial comparing 7 vs 14 days of therapy for episodes with uncomplicated candidemia and the reduction in antifungal treatment days under a hypothetical 7-day treatment strategy.

**Results:**

Among 314 episodes of candidemia, 103 (33%) were classified as uncomplicated. Of the 264 (84%) episodes surviving beyond the first 5 days without limitation of care, 30-day mortality was lower in uncomplicated than complicated candidemia (7% vs 20%; *P* = .006). Based on a hypothetical randomized non-inferiority trial comparing 7 vs 14 days of antifungal therapy, assuming an expected event rate of 25% and a non-inferiority margin of 7.5%, 1103 patients would be required. Overall, the 314 episodes received 6377 days of antifungal therapy (median, 14 days; interquartile range 13–18). A shortened 7-day treatment strategy for uncomplicated candidemia would have reduced antifungal exposure by 799 treatment days, corresponding to a 13% reduction.

**Conclusions:**

Uncomplicated candidemia accounted for only one-third of episodes, and abbreviated therapy would yield a modest reduction in antifungal exposure. The large sample size required and limited stewardship benefit suggest that a randomized trial would be challenging to conduct.

## Teaser

Among 314 candidemia episodes, uncomplicated candidemia represented 33% of cases with a lower mortality rate compared with complicated cases (7% vs 20%, *P* = .006). Application of criteria for shortened therapy in these uncomplicated episodes would have yielded modest reductions in antifungal use (13%).

Candidemia is associated with substantial morbidity and mortality despite advances in antifungal therapy [1–4]. In the absence of deep-seated infection, current international guidelines recommend a treatment duration of 14 days after the first negative blood culture [5, 6]. This duration originates from early randomized controlled trials comparing fluconazole with amphotericin B, in which a minimum treatment duration of 14 days was predefined in both study arms [5–9]. However, some observations suggest that, among patients without deep-seated infection, shorter courses of antifungal therapy may be sufficient [1, 2, 10]. Furthermore, mortality as the main outcome in trials evaluating candidemia treatment duration may not adequately reflect the impact of treatment length, since mortality in these patients could be mainly driven by host underlying conditions, such as age and comorbidities. The inclusion of infection-related outcomes, such as recurrence of infection, defined by new or persistent radiological signs of infection or new episodes of candidemia, may therefore be more appropriate [2, 3, 5–9]. This is consistent with data from Vena et al., whose propensity-score-adjusted study found no difference in relapse between shortened and standard treatment durations, supporting the use of infection-related rather than mortality endpoints in this population [2].

Recently, a comprehensive review proposed a standardized definition of uncomplicated candidemia, aiming to identify patients who might be assessed in randomized controlled trials for shorter antifungal courses [11]. Uncomplicated candidemia was defined according to a set of criteria including absence of immunosuppression or intravenous drug use, timely and adequate source control, absence of extra-abdominal deep-seated candidiasis, clearance of candidemia within 120 hours, and infection caused by echinocandin-susceptible *Candida* species [11]. The authors suggest that a safe shorter course of antifungal therapy in this subset (eg 7 days) may result in decreased antifungal exposure with subsequent lower risk of toxicity and resistance. However, this assumption raises the question of the feasibility of such randomized trial.

The primary aim of the present study was to assess the feasibility of a future randomized controlled trial comparing 7 vs 14 days of antifungal therapy in patients with uncomplicated candidemia, by estimating the proportion of episodes meeting eligibility criteria, the sample size required for a randomized non-inferiority trial, and the anticipated reduction in antifungal exposure under a shortened-therapy strategy. The secondary objective was to define the characteristics of uncomplicated vs complicated candidemia in terms of patient underlying conditions, characteristics of infection, and outcomes.

## MATERIALS AND METHODS

This retrospective observational study was conducted over a 10-year period (2015–2024) at Lausanne University Hospital (CHUV), a tertiary care hospital located in Switzerland, with 1100 acute care beds and 44 000 admissions per year. CHUV has an oncology department for hematological and solid malignancy treatment, including a unit for acute leukemia and autologous hematopoietic stem cell transplantation and chimeric antigen receptor-T cell therapies. Furthermore, our center performs renal, lung, and heart transplantation. The incidence of candidemia is estimated at approximately 1 per 10 000 patient-days [12]. The study was approved by the Ethics Committee of the Canton of Vaud (CER-VD 2021-02516).

Eligible patients were adults (≥18 years) with at least one blood culture positive for *Candida* species during the study period and absence of the patient's decline of general consent for retrospective data use. Episodes were excluded if medical records were incomplete.

Positive blood cultures were identified by the database of the Institute of Microbiology. Clinical and demographic data, comorbidities, antifungal therapy, source control measures, presence of sepsis, and site of infection were manually extracted from electronic health records by an ID consultant (MPO) using a standardized data-collection form.

Complicated and uncomplicated candidemia was defined based on the criteria proposed by Reinhold et al. as detailed in Supplementary Table 1 [11]. Sepsis and septic shock were defined according to the Sepsis-3 International Consensus criteria [13]. Appropriate antifungal therapy was defined as initiation of at least one antifungal agent with documented in vitro activity against the causative *Candida* species [4]. Source control was considered indicated in the following situations: removal of intravascular catheters in cases of catheter-related candidemia or candidemia of unknown origin when a venous catheter was in place and radiologically guided or surgical drainage of infected collections or removal of infected material [4]. The proposed definition of uncomplicated or complicated candidemia was applied only to episodes in patients who survived the first 5 days after candidemia onset and had no limitation of care, as assessment of candidemia clearance, adequacy of source control, and exclusion of deep-seated infection requires at least 5 days of follow-up [11].

To assess the feasibility of a future randomized controlled trial comparing 7 vs 14 days of antifungal therapy in patients with uncomplicated candidemia, we adopted the following approach: (1) assess the proportion of uncomplicated candidemia in a real-life clinical setting, (2) calculate the sample size for such a randomized trial to meet non-inferiority criteria, (3) and estimate the number of participating centers required to complete enrollment within 5 years under different recruitment scenarios. The proposed primary endpoint of the hypothetical randomized trial was a composite of 90-day mortality, candidemia recurrence, or need for additional source control, defined as any unplanned repeat surgical or percutaneous drainage performed after the patient's inclusion (Table 1). The expected event rate of 25% in both arms was derived from a recent study of shortened therapy in uncomplicated candidemia, which reported a combined 90-day mortality and recurrence rate of approximately 23% regardless of treatment duration (90-day mortality was 23% in the short-course group and 23% in the prolonged-course group) and a 1-year recurrence rate of 7%, most of which occurred within the first 90 days (7% in the short-duration group and 9% in the long-duration group) [2]. A non-inferiority margin of 7.5% was chosen as a compromise between feasibility considerations associated with a smaller margin and the risk of accepting a clinically unacceptable loss of efficacy [14, 15]. The one-sided alpha was set at 0.025, with 80% power and a 1:1 allocation ratio.

**Table 1.** Assumptions for Sample Size Calculation in a Non-Inferiority Trial Comparing 7 vs 14 Days of Antifungal Therapy in Uncomplicated Candidemia

Parameter	Value
Study design	Randomized, controlled, non-inferiority trial
Population	Patients with uncomplicated candidemia
Intervention	7-day antifungal therapy
Comparator	14-day antifungal therapy
Primary endpoint	Composite outcome: 90-day mortality, candidemia recurrence, or need for additional source control
Expected event rate (control group)	25%
Expected event rate (intervention group)	25%
Non-inferiority margin (Δ)	7.5%
Type I error (α)	0.025 (one-sided)
Power (1–β)	80%
Allocation ratio	1:1
Required sample size (per group)	524 patients
Total sample size	1048 patients
Estimated attrition rate	5%
Adjusted total sample size	1103 patients

Episodes with uncomplicated and complicated candidemia were compared. To estimate the potential impact of shortened antifungal therapy, we modeled a hypothetical strategy in which all patients with uncomplicated candidemia received 7 days of antifungal therapy after the first negative follow-up blood culture and calculated the corresponding reduction in total antifungal treatment days.

Statistical analyses were performed using SPSS Statistics version 26.0 (IBM Corp., Chicago, IL, USA). Categorical variables were compared using the chi-square test or Fisher's exact test, as appropriate, and continuous variables using the Mann-Whitney *U* test. All statistical tests were two-tailed, and a *P* < .05 was considered statistically significant.

## RESULTS

**Figure 1.**
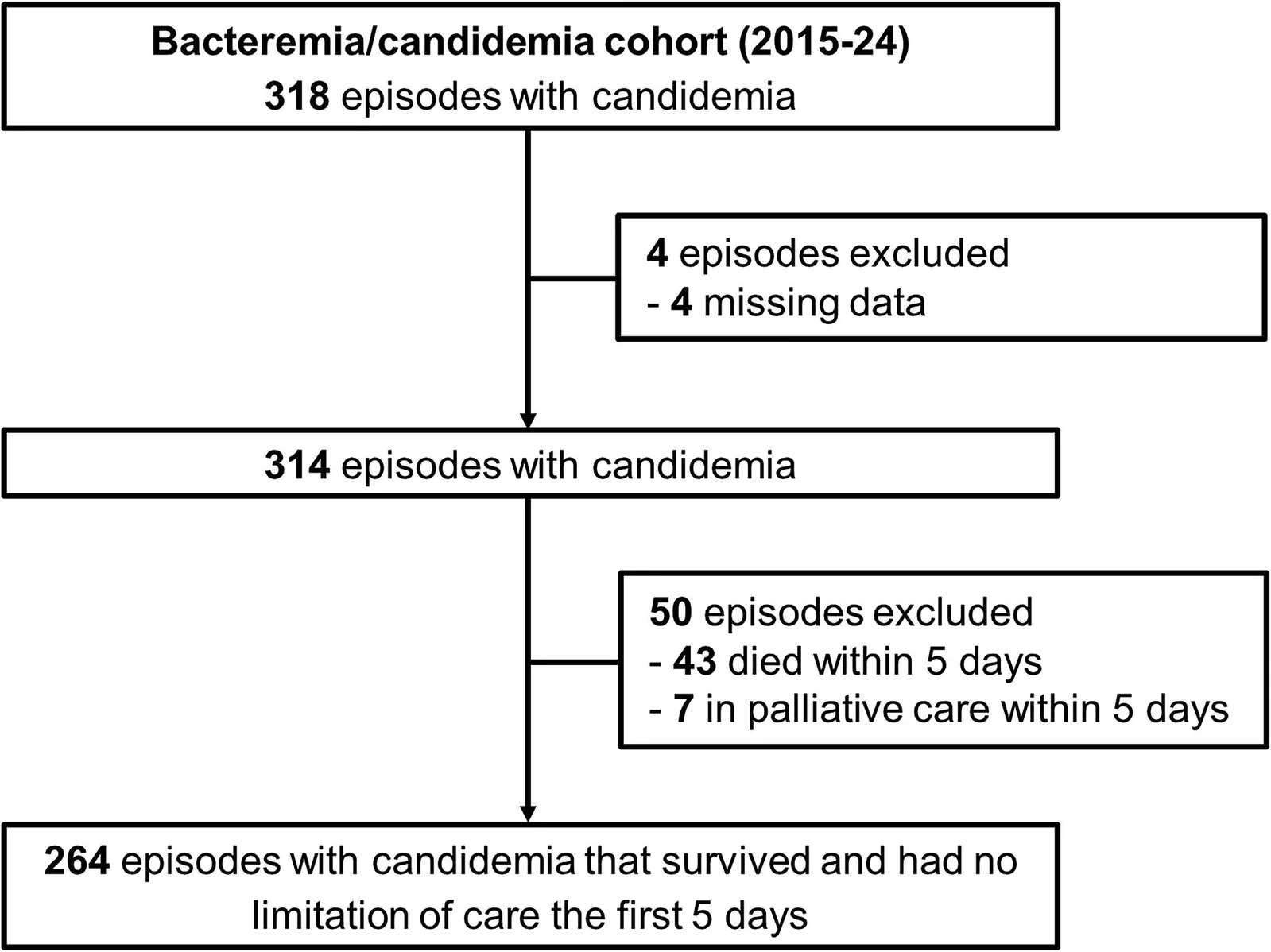
Flowchart of patients’ selection.

A total of 314 episodes of candidemia were identified in 290 patients (Figure 1); 24 episodes represented recurrent candidemia, with a median interval of 4 months between episodes (range, 1–53 months) (Supplementary Table 2). Follow-up blood cultures documenting clearance of candidemia were performed in 298 (95%) episodes. Diagnostic workup included echocardiography in 169 (54%) episodes, thoracoabdominal imaging in 201 (64%), and cerebral imaging in 60 (19%) episodes.

Catheter-related candidemia was the most common source of infection (179 episodes; 57%), followed by abdominal infections (89 episodes; 28%). Based on the proposed definition, 103 (33%) episodes were classified as uncomplicated candidemia (Table 2). Among them, 23 (22%) episodes had an infection focus warranting a treatment duration of at least 2 weeks of antifungal therapy, such as intra-abdominal abscesses managed with percutaneous drainage, tertiary peritonitis, or urinary tract infections associated with foreign bodies. *Candida albicans* was more frequent in uncomplicated cases, while non-*albicans Candida* spp., notably *Candida tropicalis*, predominated in complicated cases.

**Table 2.** Comparison of Uncomplicated and Complicated Candidemia Episodes

	Uncomplicated (*n* = 103)	Complicated (*n* = 211)	*P*
Demographics			
Male sex, *n* (%)	71 (69)	146 (69)	1.000
Age (years), median (IQR)	67 (56–76)	66 (49–74)	.208
Age >65 years, *n* (%)	56 (54)	108 (51)	.631
Comorbidities			
Malignancy (solid organ or hematologic), *n* (%)	34 (33)	81 (38)	.384
Hematological malignancy without remission^a^, *n* (%)	0 (0)	9 (4)	.033
Allogeneic hematopoietic stem cell transplantation^a^, *n* (%)	0 (0)	1 (0.5)	1.000
Immunosuppression	…	…	
Recent or ongoing use of glucocorticosteroids^a^, *n* (%)	0 (0)	31 (15)	<.001
Neutropenia^a^, *n* (%)	0 (0)	27 (13)	<.001
Diabetes mellitus, *n* (%)	30 (29)	59 (28)	.894
Chronic kidney disease (moderate or severe), *n* (%)	16 (16)	41 (19)	.439
Intravenous drug use^a^, *n* (%)	0 (0)	28 (13)	<.001
Vascular indwelling prosthetic material^a^, *n* (%)	0 (0)	42 (20)	<.001
Prosthetic intracardiac material, *n* (%)	0 (0)	29 (14)	<.001
Endovascular graft, *n* (%)	0 (0)	19 (9)	.001
Charlson Comorbidity Index (points), median (IQR)	5 (2–7)	5 (3–7)	.699
Charlson Comorbidity Index >4, *n* (%)	54 (52)	111 (53)	1.000
Microbiological data			
Type of *Candida* spp.	…	…	
*C albicans*, *n* (%)	62 (60)	101 (48)	.042
*Nakaseomyces glabratus* (*C glabrata*), *n* (%)	27 (26)	71 (34)	.197
*C tropicalis*, *n* (%)	2 (2)	20 (10)	.017
*C parapsilosis*, *n* (%)	7 (7)	6 (3)	.130
Other *Candida* spp., *n* (%)	8 (8)	22 (10)	.543
Multiple *Candida* spp., *n* (%)	3 (3)	8 (4)	1.000
At least 2 positive blood culture sets, *n* (%)	34 (33)	90 (43)	.111
Echinocandin resistance^a^, *n* (%)	0 (0)	8 (4)	.057
Persistent candidemia ≥120 hours^a^, *n* (%)	0 (0)	33 (16)	<.001
Polymicrobial infection, *n* (%)	23 (22)	54 (26)	.78
Candidemia in the prior 90 days^a^, *n* (%)	0 (0)	4 (2)	.307
Manifestations			
Community-onset, *n* (%)	19 (18)	33 (16)	.522
Fever (temperature >38°C), *n* (%)	78 (76)	166 (79)	.566
Sepsis or septic shock, *n* (%)	61 (59)	129 (61)	.806
Chorioretinitis^a^, *n* (%)	0 (0)	14 (7)	.006
Focus of infection			
Catheter-related, *n* (%)	59 (57)	120 (57)	1.000
Abdominal, *n* (%)	25 (24)	64 (30)	.288
Urinary tract, *n* (%)	16 (16)	10 (5)	.002
Bone and joint infection^a^, *n* (%)	0 (0)	5 (2)	.176
Infective endocarditis^a^, *n* (%)	0 (0)	8 (4)	.057
Other site of infection, *n* (%)	4 (4)	14 (7)	.441
Management			
Source control interventions	…	…	
All types of source control interventions	…	…	<.001
Not warranted, *n* (%)	13 (13)	23 (11)	
Warranted and performed, *n* (%)	87 (85)	137 (65)	
Warranted, but not performed, *n* (%)	3 (3)	51 (24)	
Absence of catheter removal within 48 hours from candidemia diagnosis^a,b^, *n* (%)	0 (0)	70 (58)	<.001
Absence of source control intervention within 120 hours from candidemia onset^a,c^, *n* (%)	0 (0)	33 (64)	<.001
Antifungal treatment	…	…	
Antifungal treatment initiation within 72 hours, *n* (%)	91 (88)	184 (87)	.857
Appropriate antifungal treatment within 72 hours, *n* (%)	90 (87)	177 (84)	.501
Duration of antifungal treatment (days), median (IQR)	14 (13–16)	15 (12–22)	.210
Focus of infection warranting at least 2 weeks of antifungal treatment^d^, *n* (%)	23 (22)	-	-
Intensive care unit admission^e^, *n* (%)	51 (50)	94 (45)	.470
Outcomes in whole cohort			
Mortality or limitation of care in the first 5 days, *n* (%)	15 (15)	35 (17)	.743
30-day mortality, *n* (%)	21 (20)	70 (33)	.024
90-day mortality, *n* (%)	32 (31)	88 (42)	.083
90-day candidemia recurrence, *n* (%)	2 (2)	8 (4)	.507
Outcomes among 264 candidemia episodes that survived and had no limitation of care in the first 5 days			
30-day mortality, *n* (%)	6 (7)	35 (20)	.006
90-day mortality, *n* (%)	17 (19)	53 (30)	.076
90-day candidemia recurrence, *n* (%)	2 (2)	8 (5)	.504

IQR, interquartile range.

^a^Items that are part of the definition of complicated candidemia.

^b^Among 179 episodes with catheter-related candidemia.

^c^Among 67 episodes with intra-abdominal infection warranting source control intervention.

^d^Including episodes with intra-abdominal abscesses that were treated with percutaneous drainage, episodes with tertiary peritonitis, or infections associated with foreign bodies in the urinary tract; among episodes with uncomplicated candidemia.

^e^Episodes occurring in patients who were already in the intensive care unit at candidemia onset or who were admitted to the intensive care unit within 5 days of candidemia onset.

Overall 30-day all-cause mortality was 29% (91 episodes). Care withdrawal within 5 days of candidemia onset occurred in 45 (14%) episodes. Among the 264 (84%) episodes that survived beyond the first 5 days without limitation of care, 30-day mortality was 16% (41 episodes) and was higher in episodes classified as complicated compared with uncomplicated candidemia (20% vs 7%; *P* = .006). Ninety-day mortality was 27% (70 episodes), with higher rates in complicated episodes compared with uncomplicated candidemia (30% vs 19%; *P* = .076). Ninety-day recurrence of candidemia was observed in 10 (4%) episodes, with no significant difference between complicated and uncomplicated cases (5% vs 2%; *P* = .504).

Based on the specifications of the hypothetical randomized controlled trial (Table 1), the required sample size was 524 patients per arm (1048 total). After adjusting for an anticipated 5% loss to follow-up, the total required sample size was 1103 patients. The projected number of participating centers required to complete enrollment within a feasible timeframe of 5 years enrollment period was derived directly from the incidence of eligible episodes observed in our cohort. Over the 10-year study period, our center identified 314 episodes of candidemia in adult patients. After excluding 50 episodes in patients who died within 5 days of onset or were under palliative care, 264 episodes remained, of which 103 (39%) met the criteria for uncomplicated candidemia and would therefore have been eligible for a shortened-therapy trial. This corresponds to an average of 10.3 eligible episodes per year at our institution. We used this rate as the reference incidence for extrapolating trial feasibility, under the assumption that centers of comparable size and case mix would identify eligible patients at a similar rate. Based on different assumed consent rates, we calculated the number of centers required to include 1103 patients within a 5-year enrollment period (Supplementary Table 3). Even with a moderate consent rate of 60%–70% across all centers, approximately 31–36 centers would be needed to complete enrollment within 5 years.

Episodes with complicated candidemia had a median treatment duration of 15 days (IQR: 12–2), compared with 14 days (IQR: 13–16) in uncomplicated cases. In total, 6377 days of antifungal therapy were administered across the 314 episodes (median: 14 days; IQR: 13–18 days). Under a hypothetical scenario in which patients classified as having uncomplicated candidemia received a shortened course of treatment, this would have resulted in a reduction of 799 antifungal days, corresponding to a 13% decrease. When further excluding from the uncomplicated group those episodes in which the source of infection inherently warrants at least 14 days of therapy, such as tertiary peritonitis or intra-abdominal (including hepatic) abscesses managed with percutaneous drainage, the estimated reduction decreased to 402 antifungal days (6%).

## DISCUSSION

In this retrospective cohort, only a small proportion of episodes met the criteria for uncomplicated candidemia, and a hypothetical study evaluating short-course treatment for such episodes would require a huge effort in terms of recruitment to achieve a sufficient sample size to demonstrate the non-inferiority of this approach. Moreover, we estimated that such a strategy would have resulted in a relatively modest reduction in overall antifungal use.

Overall 30-day mortality (29%) and 90-day recurrence (3%) were consistent with previous cohorts [1–4, 16, 17]. As expected, mortality was significantly lower in uncomplicated vs complicated candidemic episodes, which supports the rationale to target this population for shorter antifungal treatment courses. However, mortality in candidemia is largely driven by host factors such as age, comorbidities, and underlying disease, whereas treatment duration likely plays a limited role [2–4]. Accordingly, recurrence or persistence of infection may represent more appropriate endpoints for guiding treatment strategies [18]. This shift toward infection-related outcomes has also been proposed in the management of *Staphylococcus aureus* bacteremia [19]. Interestingly, we observed no differences in recurrence rates of candidemia, which were low (<5%) in both groups.

Interestingly, uncomplicated candidemia represented only a small proportion of cases (one-third). These patients received about 14 days of antifungal therapy, as recommended by current guidelines, which actually did not differ from patients with complicated candidemia (median 15 days) [5, 6]. Assuming these patients with uncomplicated candidemia could have received a shortened course of therapy (7 days), the estimated reduction in overall antifungal use would be modest (13%). Considering patients with infectious foci that inherently require prolonged therapy (intra-abdominal abscesses or tertiary peritonitis), this benefit would be reduced to only a 6% decrease. Source control is a key determinant of outcomes in candidemia, yet not all interventions are equivalent: surgical debridement generally provides definitive control, whereas percutaneous drainage often necessitates longer therapy [4, 11, 20–22]. In our institution, intra-abdominal abscesses managed without surgical debridement and tertiary peritonitis were treated for at least 2 weeks, frequently extending to 4–6 weeks depending on clinical and radiological response [5, 6, 20, 23]. Including such cases within uncomplicated candidemia may therefore increase the risk of treatment failure or recurrence [5, 6, 11]. While a reduction of 6%–13% in total antifungal exposure may be modest, even decreases of this magnitude may be clinically meaningful from an antifungal stewardship perspective, given their potential cumulative impact on drug-related toxicity, selection pressure for resistance, and healthcare costs [24–27].

Our results raise the question of the feasibility of a randomized controlled trial comparing 7 vs 14 days of treatment for uncomplicated candidemia. Using a composite endpoint of 90-day mortality, recurrence, and need for additional source control would require more than 1000 patients, assuming an event rate of 25% and a non-inferiority margin of 7.5%. Given the relative rarity of candidemia, such a study would require a large multicenter effort, with approximately 31–36 centers of size and case mix similar to our hospital needed to complete enrollment within 5 years, assuming a moderate consent rate (60%–70%).

This study has several limitations. First, it is a retrospective, single-center study conducted in a tertiary-care setting with systematic ID consultation and follow-up blood cultures, which may limit generalizability. Second, the number of included episodes was moderate, although this study represents, to our knowledge, the first external evaluation of the proposed definition. Third, confounding factors, including decisions to limit care, may have influenced outcomes and introduced survivorship bias; to mitigate this, analyses were restricted to patients surviving beyond 5 days without care limitation. Finally, as all patients were managed according to guideline-recommended treatment durations of at least 14 days, we were unable to directly assess the safety of shorter regimens.

In conclusion, the proposed definition of complicated candidemia identifies patients at higher risk of mortality but did not predict recurrence, which may represent a more reliable indicator of failure of therapy rather than overall mortality. Although shorter treatment strategies warrant evaluation, their overall impact on antifungal consumption is likely to remain limited even if proven safe. Moreover, a randomized controlled study to demonstrate this modest benefit raises questions about its feasibility, considering the high number of inclusions needed to reach sufficient statistical power. Therefore, further efforts for optimized definitions of uncomplicated candidemia and antifungal treatment duration would be warranted in order to maximize the feasibility and impact of such an approach.

## Supplementary Material

ofag530_Supplementary_Data

## Supplementary Data

Supplementary materials are available at *Open Forum Infectious Diseases* online. Consisting of data provided by the authors to benefit the reader, the posted materials are not copyedited and are the sole responsibility of the authors, so questions or comments should be addressed to the corresponding author.
