# Supplementary material for: Challenging the Concept of Uncomplicated Candidemia for Shorter Courses of Antifungal Therapy
Source: Open Forum Infect Dis. 2026 Aug 22;13(9):ofag530. doi: 10.1093/ofid/ofag530 (PMC13542719; doi:10.1093/ofid/ofag530)
Supplement: Supplementary file 1 [file ofag530_supplementary_data.pdf]

Supplementary Table 1. Criteria for uncomplicated candidemia as proposed by Reinhold *et al*.

**Host characteristics**
• No neutropenia (<500 cells/µl) for more than 10 days in the last 30 days
• No recent or newly initiated ongoing use of glucocorticosteroids (≥0.3 mg/kg/day for ≥3 weeks)
• No hematological malignancy without remission
• No allogeneic hematopoietic stem-cell transplantation
• No acute graft-versus-host disease grade III or IV
• Absence of active illicit intravenous drug use
**Source control**
• Central venous line, if any, is removed within 24-48h after diagnosis of candidemia
• No implantable cardiac electronic device, ventricular assist device, extracorporeal membrane oxygenation, or indwelling intravascular foreign body that cannot be removed present at onset of candidemia
• Intra-abdominal candidiasis (eg, abdominal abscess or peritonitis) is surgically or interventionally controlled
• Absence of extra-abdominal deep-seated candidiasis
• No central nervous system infection, chorioretinitis, endophthalmitis, intravascular infection, endocarditis, renal abscess, osteomyelitis, or joint infection
**Microbiological factors**
• Absence of echinocandin-resistant *Candida* species
• Candidemia less than 120h between blood collection of the index blood culture and the first negative blood culture
**Clinical response**

- No attributable signs or symptoms for persistent candidemia 120h or more after treatment initiation
- No signs, symptoms, or indicative imaging for deep-seated candidiasis 120h or more after treatment initiation

Supplementary Table 2. Diagnoses of episodes with recurrent candidemia

	Initial episode		Time between episodes (months)	Subsequent episode	
	**Species**	**Diagnosis**		**Species**	**Diagnosis**
1	*C. albicans*	Catheter-related[a]	2	*C. glabrata*	Catheter-related
2	*C. albicans*	Catheter-related[a]	2	*C. albicans*	Catheter-related
3	*C. albicans*	Catheter-related[a]	2	*C. albicans*	Catheter-related
4	*C. albicans*	Abdominal	2	*C. inconspicua*	Abdominal
5	*C. albicans*	Catheter-related[a]	2	*C. albicans*	Catheter-related
6	*C. glabrata*	Abdominal infection	2	*C. dubliniensis*	Abdominal infection
7	*C. albicans*	Catheter-related[a]	2	*C. albicans*	Infective endocarditis
8	*C. glabrata*	Catheter-related[a]	2	*C. glabrata*	Catheter-related
9	*C. albicans*	Catheter-related[a]	3	*C. albicans*	Urinary-tract
10	*C. albicans*	Catheter-related[a]	3	*C. albicans*	Spondylodiscitis
11	*C. glabrata*	Abdominal	4	*C. albicans*	Catheter-related
12	*C. inconspicua*	Abdominal	4	*C. albicans*	Catheter-related
13	*C. albicans*	Abdominal	4	*C. orthopsilosis*	Catheter-related
14	*C. tropicalis*	Catheter-related[a]	5	*C. glabrata*	Catheter-related
15	*C. albicans*	Abdominal	5	*C. glabrata*	Abdominal
16	*C. tropicalis*	Catheter-related[a]	6	*C. tropicalis*	Catheter-related

17	*C. tropicalis*	Catheter-related[a]	6	*C. glabrata*	Catheter-related
18	*C. albicans*	Catheter-related[a]	8	*C. albicans*	Catheter-related
19	*C. dubliniensis*	Catheter-related[a]	9	*C. glabrata*	Catheter-related
20	*C. pelliculosa*	Catheter-related[a]	10	*C. tropicalis*	Catheter-related
21	*C. glabrata*	Catheter-related[a]	13	*C. albicans*	Catheter-related
22	*C. glabrata*	Catheter-related[a]	22	*C. albicans*	Catheter-related
23	*C. tropicalis*	Catheter-related[a]	26	*C. tropicalis*	Catheter-related
24	*C. lusitaniae*	Abdominal	53	*C. tropicalis*	Catheter-related

[a]Catheter was removed

Species names are reported using the conventional clinical nomenclature; current taxonomic names include *Nakaseomyces glabratus* (*C. glabrata*), *Clavispora lusitaniae* (*C. lusitaniae*), *Pichia cactophila* (*C. inconspicua*), and *Wickerhamomyces anomalus* (*C. pelliculosa*).

Supplementary Table 3. Estimated number of participating centers required to complete enrollment of 1103 patients within 5 years, by assumed consent rate

Consent rate (assumption)	Enrolled/center/year	Centers needed[a]
50% (conservative)	5.15	~43 centers
60%	6.18	~36 centers
70% (moderate)	7.21	~31 centers
85% (optimistic)	8.76	~25 centers

[a]assuming centers are of comparable size and case-mix to that of Lausanne University Hospital (CHUV)
